# Cerebrolysin as an adjunct to speech-language therapy in post-stroke Wernicke’s aphasia: a case report

**DOI:** 10.25122/jml-2026-0057

**Published:** 2026-06

**Authors:** Marina Romano

**Affiliations:** 1 Department of Neurology, CEMIC, Buenos Aires, Argentina

**Keywords:** stroke, rehabilitation, treatment

## Abstract

Post-stroke aphasia is a debilitating condition affecting approximately one-third of stroke survivors, profoundly impairing communication, social participation, and quality of life. While speech-language therapy (SLT) remains the standard of care, pharmacological agents such as Cerebrolysin may enhance recovery through neuroprotection and neuroplasticity. We report the case of a 38-year-old female lawyer who developed acute Wernicke’s aphasia and right-sided hemiparesis following an ischemic stroke. Despite timely thrombolysis and mechanical thrombectomy, she experienced significant neurological deficits. Early multidisciplinary rehabilitation, combined with a 10-day course of Cerebrolysin, memantine, and sertraline, was associated with substantial improvements in language, motor function, and independence over a three-month period. This case highlights the potential synergistic effect of Cerebrolysin in a young patient with early reperfusion and intensive rehabilitation, including pharmacotherapy and SLT, especially in settings where optimal post-stroke rehabilitation resources may be limited.

## Introduction

Aphasia is one of the most disabling consequences of stroke, affecting roughly one-third of survivors [1]. Clinical presentations vary widely, from difficulty retrieving words to complete loss of speech, comprehension, reading, or writing abilities. These deficits profoundly impact daily functioning, social interaction, employment, and psychological well-being [2]. Recovery is most rapid within the first 3 to 6 months, yet many patients continue to experience residual deficits despite standard care [3].

Speech-language therapy remains the cornerstone of aphasia rehabilitation. However, pharmacological agents such as memantine [4] and donepezil [5] have demonstrated limited efficacy [6]. Cerebrolysin, a neurotrophic peptide preparation, has been shown to promote neurogenesis and synaptogenesis, facilitating rewiring of language networks after ischemic injury. Evidence suggests that combining Cerebrolysin with speech-language therapy (SLT) may enhance functional recovery, as demonstrated in the Efficacy and Safety of Cerebrolysin in the Treatment of Aphasia After Acute Ischemic Stroke (ESCAS) trial [3].

Diagnosing and managing aphasia is particularly challenging in low- and middle-income countries (LMICs). Limited access to trained speech-language pathologists, delayed presentation to the hospital, lack of standardized assessment tools, and insufficient rehabilitation infrastructure can impede timely intervention, reducing the potential for optimal recovery. Pharmacological adjuncts that enhance neuroplasticity may therefore be particularly valuable in such contexts [6]. Only 1-5% of the Buenos Aires population has real access to all reperfusion therapies [7].

## Case presentation

A 38-year-old female lawyer with no significant past medical history presented with sudden-onset Wernicke’s aphasia and severe right-sided hemiparesis while moving houses. She arrived at the hospital 2 hours after symptom onset. Initial assessment revealed a score of 21 on the National Institutes of Health Stroke Scale (NIHSS), and fluent Wernicke’s aphasia was diagnosed. Her pre-stroke modified Rankin Scale (mRS) score was 0, indicating full functional independence.

### Diagnostic assessment

**Figure 1:**
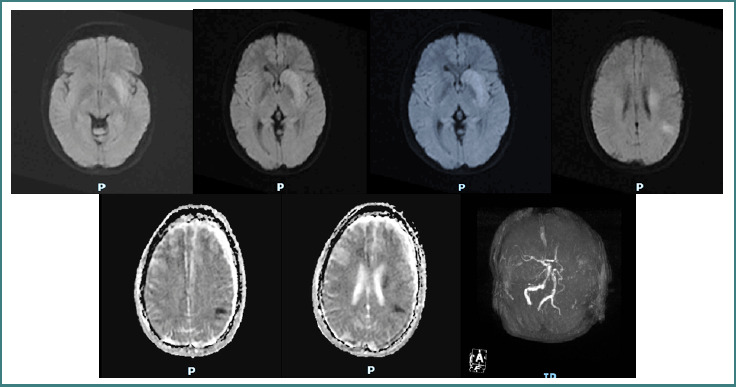
Brain MRI at acute presentation. Axial diffusion-weighted imaging (DWI) sequence demonstrating areas of restricted diffusion in the left basal ganglia and ipsilateral parietal cortex (Wernicke area), consistent with acute ischemic lesions.

Brain MRI (Figure 1) revealed restricted diffusion in the left basal ganglia and ipsilateral parietal cortex (Wernicke area), consistent with acute ischemic lesions. Intracranial imaging showed absent flow in the left internal carotid artery and middle cerebral artery, confirming large-vessel occlusion. Prompt imaging and diagnosis allowed initiation of reperfusion therapy within the recommended window, which is often a challenge in LMICs due to delays in access and diagnostic limitations [7,8,9].

### Therapeutic intervention

The patient received intravenous alteplase (recombinant tissue plasminogen activator [rt-PA]) followed by emergency mechanical thrombectomy. Two stents were successfully placed in the left internal carotid artery, achieving Thrombolysis in Cerebral Infarction (TICI) grade IIc reperfusion. Antiplatelet therapy included tirofiban and clopidogrel. During hospitalization, she developed decreased consciousness due to hemorrhagic transformation; tirofiban was discontinued, and no neurosurgical intervention was required. She remained on mechanical ventilation for 5 days, after which she was successfully extubated.

**Figure 2:**
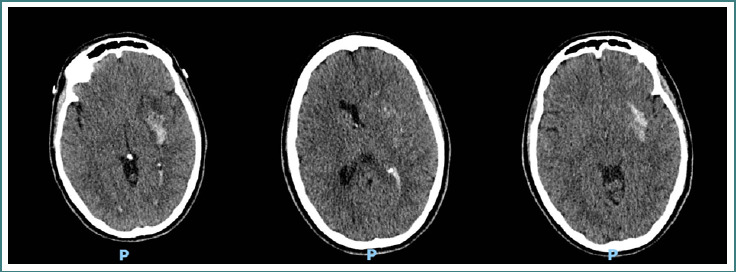
CT scan after extubation on day 5 with hemorrhagic transformation

At extubation, she was alert and oriented, with moderate residual hemiparesis, dysphagia, and mild aphasia. Clinical assessments demonstrated an NIHSS score of 17, a mRS score of 3, a Western Aphasia Battery (WAB) score of 37, and a Barthel Index (BI) score of 35, indicating substantial neurological impairment and severe functional dependency (see Supplementary File S1 for details regarding these assessment scales) (Figure 2).

Supplementary File

### Rehabilitation and pharmacological intervention

The patient was transferred to a neurological rehabilitation facility with a nasogastric tube, aphasia, hemiparesis, and mild post-stroke depression. She participated in a multidisciplinary rehabilitation program including physical therapy, occupational therapy, SLT, and psychological support.

The rehabilitation program, which is often difficult to access in LMICs, consisted of physical therapy sessions twice a day, occupational therapy three times a week, SLT for 1 h, four times a week, and psychological support once a week.

Pharmacological therapy included a 10-day course of Cerebrolysin (30 mL/day), sertraline 50 mg/day, and memantine 20 mg/day [3,10].

After 30 days, her aphasia had significantly improved (the patient was able to understand one-step orders), the nasogastric tube was removed following a successful swallowing assessment, and she began ambulating with assistance. Clinical assessments showed an NIHSS score of 14, an mRS score of 3, a WAB score of 45, and a BI score of 52. The NIHSS score was 14, although this value was disproportionately elevated due to high subscores in the visual and sensory deficits domains.

**Figure 3:**
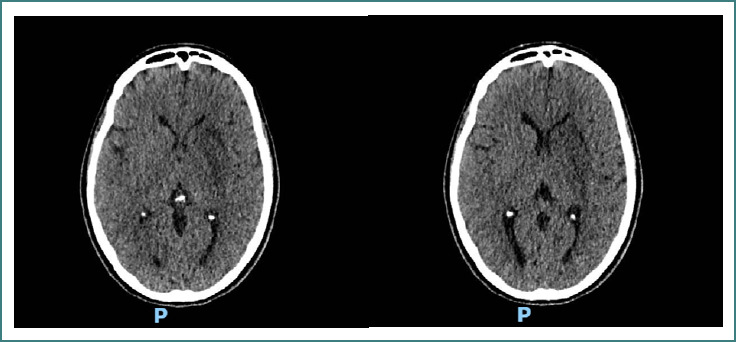
Brain CT scan at three-month follow-up

At three-month follow-up, the patient achieved independent ambulation and near-complete resolution of dysphagia. Residual deficits included minimal aphasia and mild hypophonia. Clinical assessments showed an NIHSS score of 6, an mRS score of 1, a WAB score of 69, and a BI score of 79, reflecting substantial functional recovery (Table 1 and Figure 3).

**Table 1:** Clinical and functional outcomes over time (NIHSS, mRS, WAB, BI)

Time Point	NIHSS	mRS	WAB	BI
Admission	21	0	37	35
30 days	14	3	45	52
3 months	6	1	69	79

## Discussion

This case illustrates that even with optimal acute stroke care, including thrombolysis, thrombectomy, and stenting, severe aphasia may persist, necessitating intensive rehabilitation. Aphasia profoundly impacts communication, social engagement, and the ability to return to work.

In LMICs, challenges in aphasia care are magnified. Many patients experience delays in clinical presentation, limited access to diagnostic imaging and acute treatment [7], and a scarcity of trained speech-language pathologists. Rehabilitation facilities are often under-resourced, and standardized assessments may be unavailable, making it difficult to monitor progress or tailor interventions. Pharmacological agents that enhance neuroplasticity, such as Cerebrolysin, may help bridge these gaps when combined with available SLT, improving functional outcomes [3].

Clinical recovery of speech fluency and understanding in this patient is likely to be compensated by the right hemisphere’s homologous language regions following severe degradation of the left temporal network. Some case reports reinforce the critical role of right-hemisphere plasticity in recovery and suggest that future clinical approaches could focus on targeted therapeutic stimulation, such as repetitive transcranial magnetic stimulation (rTMS), of right-hemisphere pathways to optimize language rehabilitation. It is also important to highlight the value of advanced functional perfusion imaging, such as brain single-photon emission computed tomography (SPECT), especially when standard structural imaging modalities like MRI or computed tomography (CT) appear negative. Such functional techniques may help identify underlying metabolic or perfusion abnormalities that are not visible on structural scans, thereby enabling more targeted neurorehabilitation strategies. In our case, it would have been very interesting to perform functional imaging to better understand the complex rehabilitation process [11-13].

In patients presenting with severe acute ischemic stroke (typically defined by a high NIHSS score and large vessel occlusion), mechanical thrombectomy (MT) has become the gold standard of care. According to high-quality clinical evidence, the percentage of patients achieving a functionally independent outcome, defined as an mRS score of 0–2 at 90 days, is approximately 45%-50%.

The landmark HERMES meta-analysis, which pooled data from pivotal trials such as MR CLEAN, ESCAPE, and REVASCAT, demonstrated that 46% of patients treated with MT achieved functional independence, compared to only 26.5% in the control group receiving best medical management alone [6].

Cerebrolysin is believed to facilitate cerebroprotection [6], neurogenesis, and synaptogenesis, supporting the rewiring of language networks after ischemic injury. In this patient, the addition of early multidisciplinary rehabilitation appeared to accelerate recovery, resulting in significant improvements in language, motor function, and independence. These findings align with previous studies, including the ESCAS trial, which reported enhanced outcomes when Cerebrolysin was combined with SLT [3].

## Conclusion

This case highlights the tailored multidisciplinary treatment (including early reperfusion thrombolysis plus thrombectomy, intensive rehabilitation, concomitant pharmacotherapy, memantine, and sertraline) and the potential of Cerebrolysin as an adjunct to early neurorehabilitation in severe post-stroke aphasia. When combined with structured SLT, it can significantly enhance recovery in language, motor function, and independence, especially in young patients. The case also underscores the need for tailored strategies in countries and regions where limited access to rehabilitation resources may hinder optimal recovery. Early, intensive, and multidisciplinary intervention remains critical to achieving the best functional outcomes and improving quality of life for stroke survivors.
