# Supplementary material for: Cerebrolysin as an adjunct to speech-language therapy in post-stroke Wernicke’s aphasia: a case report
Source: J Med Life. 2026 Jun;19(6):492–5. doi: 10.25122/jml-2026-0057 (PMC13474394; doi:10.25122/jml-2026-0057)
Supplement: Supplementary file 1 [file JMedLife-19-492-s001.pdf]

This supplementary appendix provides comprehensive criteria, classification thresholds, and clinical significance parameters for the standardized clinical scales used to evaluate the patient throughout the clinical course described in the accompanying case report.

1. NATIONAL INSTITUTES OF HEALTH STROKE SCALE (NIHSS)

The NIHSS is a standardized, prospective clinical instrument used by healthcare providers to objectively measure and quantify the severity of neurological deficits in patients experiencing an acute ischemic stroke [14].

- **Score range:** 0 to 42
  - Minimum Score: 0 (indicates a normal neurological examination).
  - Maximum Score: 42 (indicates severe, widespread neurological impairment).
- **Clinical significance:** The total score assists clinicians in predicting long-term patient outcomes, guiding acute therapeutic choices (e.g., reperfusion therapies), and monitoring serial changes in neurological status. Higher baseline scores are strongly associated with larger cerebral infarct volumes and a lower probability of excellent functional recovery.

Table S1. NIHSS score range and classification severity

Score range / Value	Severity / Functional classification
0–1	Normal or minor stroke
2–4	Mild stroke
5–15	Moderate stroke
16–20	Moderate to severe stroke
21–42	Severe stroke

2. WESTERN APHASIA BATTERY (WAB)

The WAB is a comprehensive, standardized diagnostic instrument designed to evaluate the linguistic and non-linguistic deficits in patients with aphasia, establish baseline skills, and identify specific diagnostic typologies [15].

- **Score range:** 0 to 100 (Aphasia Quotient)
  - Minimum Score: 0 (indicates complete loss of functional language capabilities).
  - Maximum Score: 100 (indicates language performance within standard normal limits).
- **Clinical significance:** The composite score, known as the Aphasia Quotient (AQ), determines the objective severity of the linguistic impairment and facilitates classification of the specific structural type of aphasia. Clinicians and clinical trials utilize these values to construct targeted speech-language therapy regimens and track individual recovery patterns over time.

Table S2. WAB score range and classification severity

Score range / Value	Severity / Functional classification
0–25	Very severe aphasia
26–50	Severe aphasia
51–75	Moderate aphasia
76–93.7	Mild aphasia
93.8–100	Within normal limits

3. MODIFIED RANKIN SCALE (MRS)

The mRS is a globally accepted, clinician-reported assessment tool used to measure the degree of disability, functional dependency, and handicap in patients' daily activities following a cerebrovascular event [16].

- **Score range:** 0 to 6
  - Minimum Score: 0 (indicates a total absence of neurological symptoms or residual functional deficits).
  - Maximum Score: 6 (indicates death).
- **Clinical significance:** Unlike deficit-specific neurological metrics, the mRS focuses on the patient's capacity to maintain personal independence in everyday life rather than on isolated impairment parameters.

Table S3. mRS score range and classification severity

Score range / Value	Severity / Functional classification
0	No symptoms at all
1	No significant disability; able to carry out all usual duties and activities
2	Slight disability; unable to carry out all previous activities, but able to look after own affairs without assistance
3	Moderate disability; requiring some help, but able to walk without assistance
4	Moderately severe disability; unable to walk without assistance and unable to attend to own bodily needs without assistance
5	Severe disability; bedridden, incontinent, and requiring constant nursing care and attention
6	Dead

© 2026 by the authors. This article is an open access article distributed under the terms and conditions of the Creative Commons Attribution (CC BY 4.0) license.

4. BARTHEL INDEX (BI)

The BI is an objective, performance-based scoring instrument utilized to systematically measure a patient's level of independence and performance across 10 core activities of daily living (ADLs), especially within neurological rehabilitation frameworks [17].

- **Score range:** 0 to 100
  - Minimum Score: 0 (represents total dependence on physical assistance for all assessed ADLs).
  - Maximum Score: 100 (indicates complete independence in physical functioning).
- **Clinical significance:** The final cumulative score quantifies the actual amount of physical assistance the patient requires. The index systematically monitors 10 functional variables: feeding, bathing, grooming, dressing, bowel control, bladder control, toilet use, transfers (bed-to-chair), mobility (on level surfaces), and stair climbing.

Table S4. BI score range and classification severity

Score range / Value	Severity / Functional classification
0–20	Total dependence
21–60	Severe dependence
61–90	Moderate dependence
91–99	Slight dependence
100	Complete independence

REFERENCES

14 Lyden P, Raman R, Liu L, Emr M, Warren M, Marler J. National Institutes of Health Stroke Scale certification is reliable across multiple venues. Stroke. 2009 Jul;40(7):2507-11. doi: 10.1161/STROKEAHA.108.532069

15. Kertesz, A. Western Aphasia Battery-Revised (WAB-R) [Database record]. APA PsycTests. 2006. doi: 10.1037/t15168-000

16. van Swieten JC, Koudstaal PJ, Visser MC, Schouten HJ, van Gijn J. Interobserver agreement for the assessment of handicap in stroke patients. Stroke. 1988 May;19(5):604-7. doi: 10.1161/01.str.19.5.604

17 Mahoney FI, Barthel DW. Functional evaluation: the Barthel Index. Md State Med J. 1965 Feb;14:56-61.

© 2026 by the authors. This article is an open access article distributed under the terms and conditions of the Creative Commons Attribution (CC BY 4.0) license.
